# Anti-inflammation of isoliquiritigenin via the inhibition of NF-κB and MAPK in LPS-stimulated MAC-T cells

**DOI:** 10.1186/s12917-022-03414-1

**Published:** 2022-08-19

**Authors:** Manman Li, Guicong Lu, Xiao Ma, Ruihong Wang, Xihong Chen, Yongxiong Yu, Caode Jiang

**Affiliations:** Chongqing Engineering Research Centre for Herbivores Resource Protection and Utilization, College of Animal Science and Technology, Southwest University, No. 2 Tiansheng Road, Chongqing, 400715 China

**Keywords:** Isoliquiritigenin, anti-inflammation, bovine mastitis, NF-κB, MAPK

## Abstract

**Background:**

The application of plant extracts has received great interest for the treatment of bovine mastitis. Isoliquiritigenin (ISL) is a rich dietary flavonoid that has significant antioxidative, anti-inflammatory and anticancer activities. This study was conducted to explore the protective efficacy and related mechanism of ISL against lipopolysaccharide (LPS)-stimulated oxidation and inflammation in bovine mammary epithelial cells (MAC-T) by *in vitro* experiments.

**Results:**

Real-time PCR and ELISA assays indicated that ISL treatment at 2.5, 5 and 10 μg/mL significantly reduced the mRNA and protein expression of the oxidative indicators cyclooxygenase-2 and inducible nitric oxide synthase (*P* < 0.01), and of the inflammatory cytokines interleukin-6 (*P* < 0.05), interleukin-1β (*P* < 0.01) and tumor necrosis factor-α (*P* < 0.01) in LPS-stimulated MAC-T cells. Moreover, Western blotting and immunofluorescence tests indicated that the phosphorylation levels of nuclear factor kappa (NF-κB) p65 and the inhibitor of NF-κB were significantly decreased by ISL treatment, thus blocking the nuclear transfer of NF-κB p65. In addition, ISL attenuated the phosphorylation levels of p38, extracellular signal-regulated kinase and c-jun NH2 terminal kinase.

**Conclusions:**

Our data demonstrated that ISL downregulated the LPS-induced inflammatory response in MAC-T cells. The anti-inflammatory and antioxidative activity of ISL involves the NF-κB and MAPK cascades.

**Supplementary Information:**

The online version contains supplementary material available at 10.1186/s12917-022-03414-1.

## Background

Bovine mastitis is considered the most costly disease in dairy cattle due to inflammation of the mammary gland. Bovine mastitis is caused by infection with microbial pathogens and physical, environmental and genetic factors as well [1]. *Escherichia coli* is one of the most common agents of clinical mastitis. Lipopolysaccharide (LPS) is the major component of the outer membrane in *Escherichia coli* [2], and it activates the nuclear factor-κB (NF-κB) pathway via Toll-like receptor 4 (TLR4) dimerization [3]. Mitogen-activated protein kinases (MAPKs) are also fundamental in the control of the inflammatory response through the crosstalk with the NF-κB pathway [3]. Both MAPK and NF-κB activation promote the production of proinflammatory factors IL-1β, IL-6 and TNF-α as well as inflammatory mediators cyclooxygenase-2 (COX-2) and inducible nitric oxide synthase (iNOS) [4, 5].

Antibiotics are widely applied in mastitis treatment; however, the abuse of antibiotics results in drug-resistant bacteria and antibiotic residues in the food chain that are harmful to consumers’ health [6]. As plant active ingredients are nontoxic and have medicinal and health benefits, their application in inflammation treatment has received widespread attention.

Isoliquiritigenin (ISL) is a flavonoid with a chalcone structure extracted from licorice. ISL has been reported to have pharmacological activities, including antioxidation, anti-inflammation, antiplatelet aggregation and antineoplastic properties [7, 8]. *In vitro* experiments have demonstrated that ISL attenuates the inflammatory response of macrophages by suppressing the homodimerization of TLR4 [9]. *In vivo* studies revealed that ISL inhibited NF-κB activation in septic mice, thus reducing the expression of IL-6, TNF-α and COX-2 [10, 11]. Additionally, upstream signaling pathways, including the phosphorylation of p38 in the MAPK pathway and DNA binding of NF-κB p65, were prohibited by ISL [12].

Despite these encouraging studies, it is still unclear whether ISL has a beneficial function in LPS-induced bovine mastitis. Here, we investigated the potential preventive effects of ISL in LPS-induced bovine mammary epithelial cells (MAC-T). In particular, we determined the molecular mechanism underlying the antioxidative and anti-inflammatory effects of ISL.

## Results

### Cytotoxicity of ISL in MAC-T cells

**Fig. 1:**
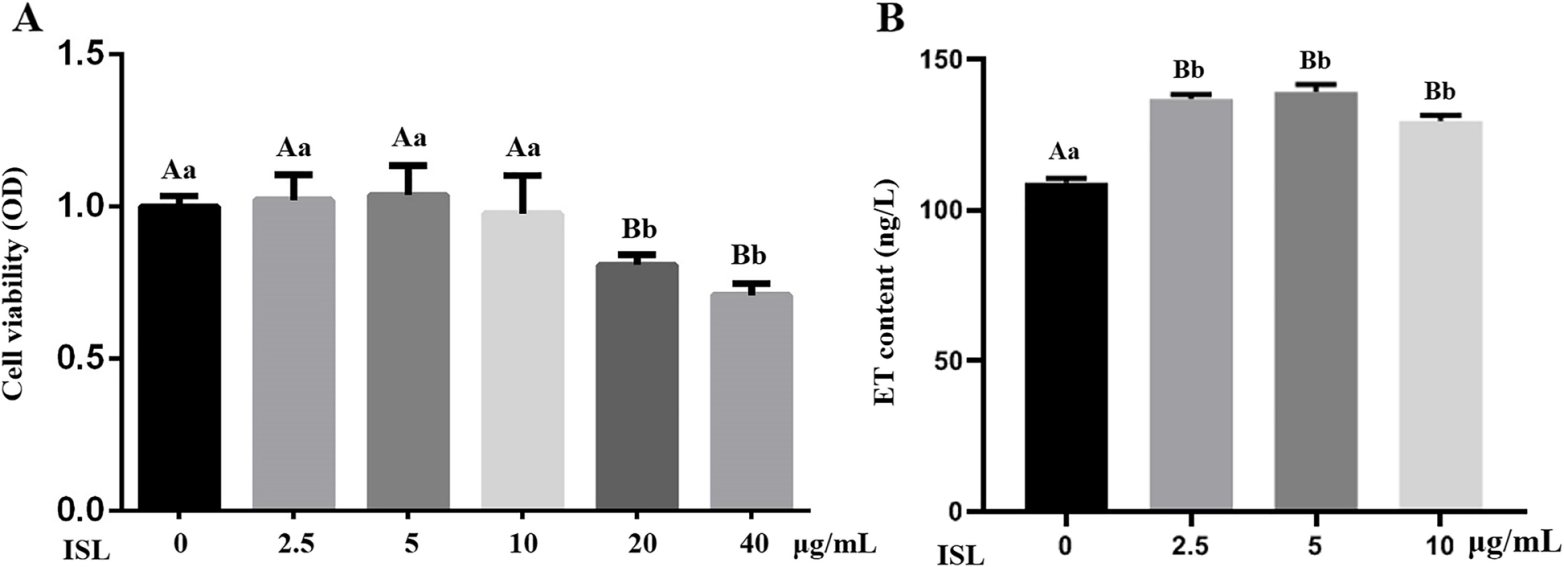
Viability and endotoxin test of MAC-T cells by MTT assay after 24 h of treatment with isoliquiritigenin (ISL). **A** Cell viability was measured using the MTT assay, where the viability of nontreatment control cells was set as 100%. **B** Cell endotoxin (ET) was tested using ELISA. Values represent the means ± SEM of three independent experiments. In the above bars, the same letters indicate *P* > 0.05; different lowercase letters indicate *P* < 0.05 while different uppercase letters indicate *P* < 0.01

The cytotoxicity of ISL in MAC-T cells was examined by MTT assay. ISL had no effect on the viability of MAC-T cells at concentrations of 2.5, 5 and 10 μg/mL; however, cell viability was significantly decreased upon 24 h treatment with ISL at 20 and 40 μg/mL (*P* < 0.05, Fig. 1A). Thus, ISL concentrations of 2.5, 5 and 10 μg/mL, which showed no cytotoxicity towards MAC-T cells, were applied in further studies. To demonstrate that there was no endogenous endotoxin present and only the effects of ISL on MAC-T cells, endogenous endotoxin was tested in the cells administered ISL at 0, 2.5, 5, 10 and 20 μg/mL (Fig.1B). The results showed much lower levels of endotoxin in the blank group than in the ISL groups (*P* < 0.01), and the endotoxin level in each cell group was below the threshold of cytotoxic response (< 200 ng/L), indicating that the plant compound is free of endotoxin.

### Anti-inflammatory and antioxidative effects of ISL in MAC-T cells

The inflammatory effect stimulated by LPS in MAC-T cells was measured using the mRNA levels of IL-6. IL-6 mRNA levels were significantly higher in LPS-treated cells than in controls (*P* < 0.01), but there was no difference in IL-6 mRNA levels upon treatment with 1, 5, 10 and 20 μg/mL LPS (*P* > 0.05, Supplementary Fig. 1). Therefore, 1 μg/mL LPS was applied for further studies [3, 13].

**Fig. 2:**
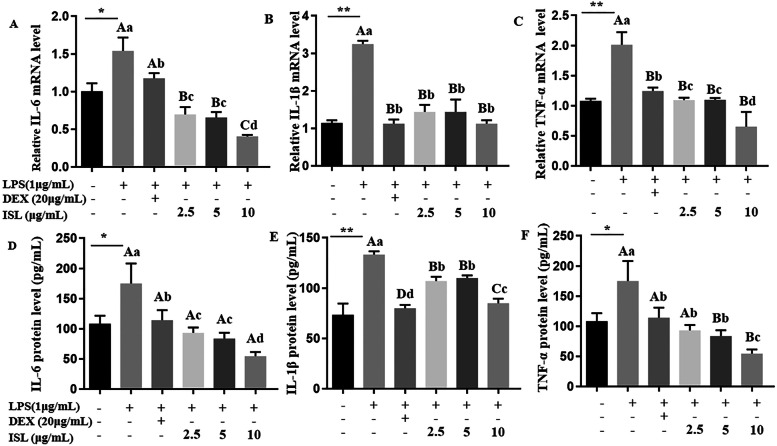
Expression analysis of proinflammatory cytokines in MAC-T cells. Cells were treated with lipopolysaccharide (LPS) at 1 μg/mL for 24 h in combination with dexamethasone (DEX) (20 μg/mL) or isoliquiritigenin (ISL) (2.5, 5 and 10 μg/mL). With β-actin as an endogenous control, real-time PCR and ELISA were used to detect mRNA (**A**-**C**) and protein (**D**-**F**) levels. Values represent the means ± SEM of four independent experiments. In the above bars, ** indicate significance at *P* < 0.01 between the control and the LPS treatment without DEX and ISL. Among LPS in combination with DEX and ISL treatments, the same letters indicate *P* > 0.05; different lowercase letters indicate *P* < 0.05 while different uppercase letters indicate *P* < 0.01

Furthermore, real-time PCR and ELISA were used to detect the expression of inflammatory markers and the inhibitory effect of ISL on inflammation. The expression of TNF-α, IL-1β and IL-6 at the mRNA and protein levels was significantly upregulated in the LPS-treated cells compared with the sham-treated cells (*P* < 0.01, Fig. 2). When ISL was administered, the mRNA levels of TNF-α (*P* < 0.01), IL-1β (*P* < 0.01) and IL-6 (*P* < 0.05) was significantly decreased, and ISL had better effects at 10 μg/mL than DEX (Fig. 2A-C). Importantly, ISL significantly inhibited the protein expression of these inflammatory biomarkers in LPS-stimulated MAC-T cells (*P* < 0.01, Fig. 2D-F), supporting the results of qRT-PCR experiments.

**Fig. 3:**
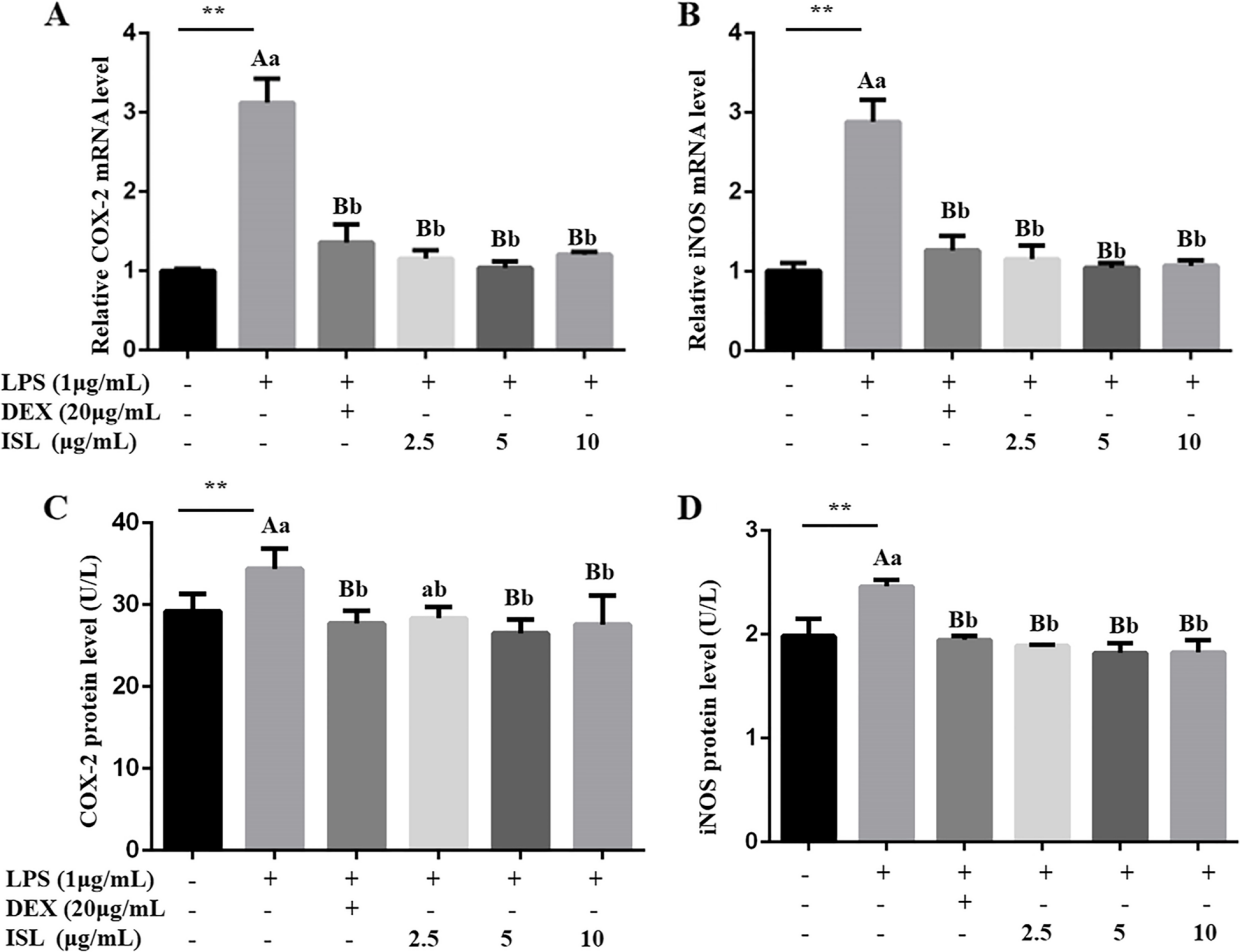
Expression analysis of COX-2 and iNOS in MAC-T cells. Cells were incubated with 1 μg/mL lipopolysaccharide (LPS) in combination with 20 μg/mL dexamethasone (DEX) or isoliquiritigenin (ISL) at 2.5, 5 and 10 μg/mL for 24 h. With β-actin as an endogenous control, real-time PCR and ELISA were used to detect mRNA (**A** and **B**) and protein (**C** and **D**) levels, respectively. Values represent the means ± SEM of four independent experiments. In the above bars, ** indicate significance at *P* < 0.01 between the control and the LPS treatment without DEX and ISL. Among LPS in combination with DEX and ISL treatments, the same letters indicate *P* > 0.05; different lowercase letters indicate *P* < 0.05 while different uppercase letters indicate *P* < 0.01

In addition, we investigated ISL influence on the expression of iNOS and COX-2, which have been reported as oxidative stress indicators. The results showed that both DEX and ISL significantly reduced LPS-induced iNOS and COX-2 mRNA abundance in MAC-T cells (*P* < 0.01, Fig. 3A-B). ELISA also showed the inhibitory effect of ISL on iNOS and COX-2 expression in LPS-stimulated MAC-T cells (Fig. 3C-D).

### Effects of ISL on LPS-stimulated activation of MAPK and NF-κB pathways

**Fig. 4:**
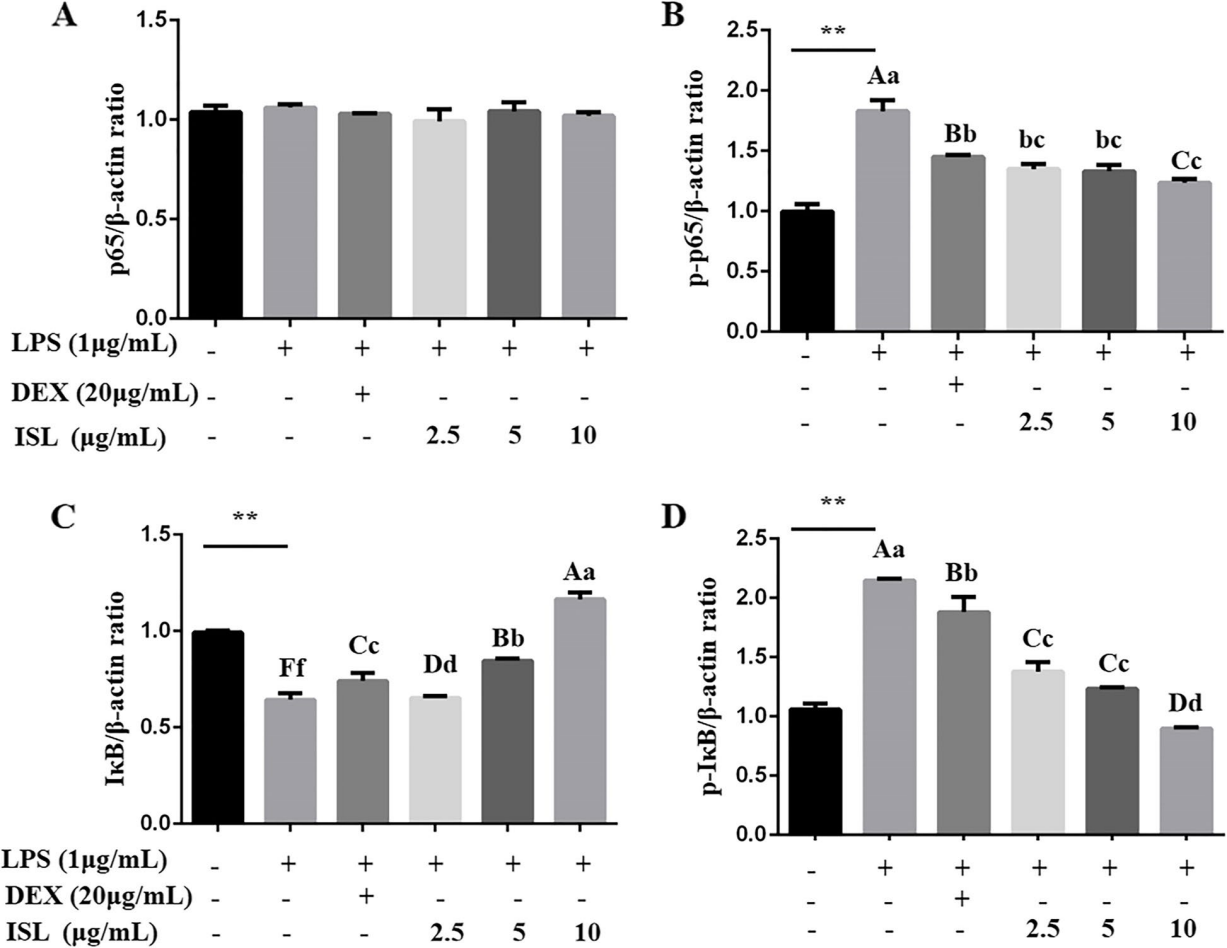
Detection of NF-κB activity in MAC-T cells by Western blotting. Cells were coincubated with 1 μg/mL lipopolysaccharide (LPS) and dexamethasone (DEX) (20 μg/mL) or isoliquiritigenin (ISL) (2.5, 5 and 10 μg/mL) for 24 h. The levels of p65 (**A**), p-p65 (**B**), IκB (**C**) and p-IκB (**D**) proteins were relative to β-actin. Values represent the means ± SEM of three independent experiments. In the above bars, ** indicate significance at *P* < 0.01 between the control and the LPS treatment without DEX and ISL. Among LPS in combination with DEX and ISL treatments, the same letters indicate *P* > 0.05; different lowercase letters indicate *P* < 0.05 while different uppercase letters indicate *P* < 0.01

We estimated how the NF-κB pathway, which is critical for the production of proinflammatory cytokines, was influenced. As shown in Fig. 4A-B and Supplementary Fig. 2A, the protein levels of p65, which is a key member in the NF-κB pathway, did not differ between LPS, DEX and ISL treatment, but the levels of phosphorylated p65 (p-p65) was significantly decreased in cells treated with ISL (2.5, 5 and 10 μg/mL) and DEX (*P* < 0.01). Moreover, ISL at 5 and 10 μg/mL increased the protein levels of IκB, which is another key member of the NF-κB pathway (*P* < 0.01, Fig. 4C). In addition, ISL markedly inhibited p-IκB levels increased by LPS (Fig. 4D) and dose-dependently decreased the p-p65/p65 and p-IκB/IκB ratios (*P* < 0.01, Supplementary Fig. 2B-C).

**Fig. 5:**
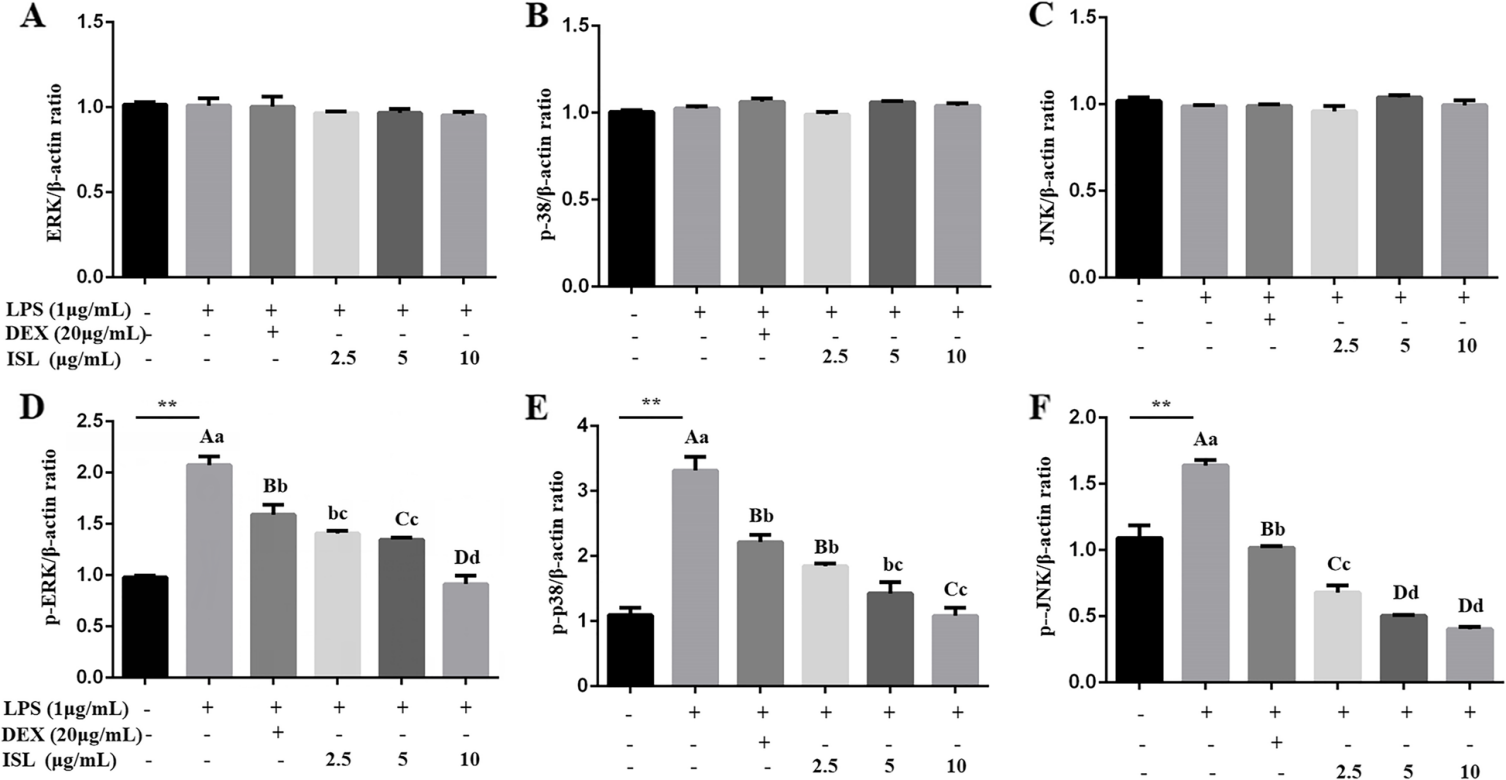
Detection of MAPK activity in MAC-T cells using Western blotting. Cells were coincubated with 1 μg/mL lipopolysaccharide (LPS) and dexamethasone (DEX) (20 μg/mL) or isoliquiritigenin (ISL) (2.5, 5 and 10 μg/mL) for 24 h. (A-F) The protein levels of ERK1/2, p38 , JNK, p-ERK, p-p38 and p-JNK were relative to those of β-actin, respectively. Values represent the means ± SEM of three independent experiments. In the above bars, ** indicate significance at *P* < 0.01 between the control and the LPS treatment without DEX and ISL. Among LPS in combination with DEX and ISL treatments, the same letters indicate *P* > 0.05; different lowercase letters indicate *P* < 0.05 while different uppercase letters indicate *P* < 0.01

We further determined the phosphorylation of MAPK members, critical cascades upstream of proinflammatory mediators and NF-κB. ISL administration significantly reduced LPS-induced activation of p-JNK, p-p38, and p-ERK (*P* < 0.01), as revealed by both their phosphorylation/total protein ratios (*P* < 0.01, Supplementary Fig. 3) and their phosphorylation/β-actin ratios (Fig. 5).

### Effect of ISL on LPS-stimulated p65 nuclear translocation

**Fig. 6:**
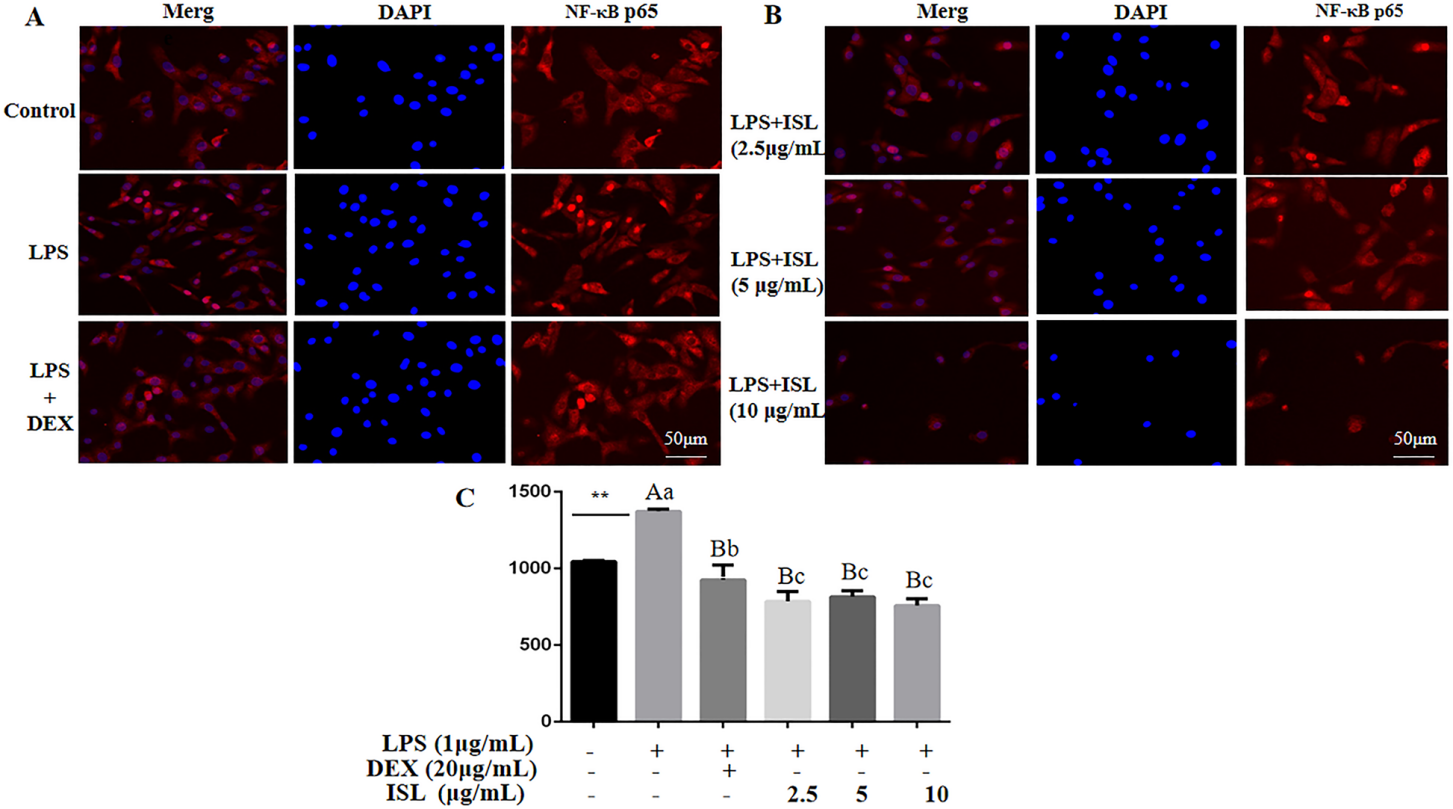
Immunofluorescence analysis (A) and quantification (B) of p65 nuclear translocation in MAC-T cells. Cells treated with LPS (1 μg/mL) were coincubated with DEX (20 μg/mL) or ISL (2.5, 5 and 10 μg/mL) for 24 h. p-p65 was labeled red with Cy3, while the nucleus was marked blue with DAPI. Values represent the means ± SEM of four independent experiments. In the above bars, ** indicate significance at *P* < 0.01 between the control and the LPS treatment without DEX and ISL. Among LPS in combination with DEX and ISL treatments, the same letters indicate *P* > 0.05; different lowercase letters indicate *P* < 0.05 while different uppercase letters indicate *P* < 0.01

Finally, we examined the nuclear translocation of NF-κB p65 in MAC-T cells challenged with LPS using an inverted fluorescence microscope. In contrast to the uniform distribution of red fluorescently labeled p65 in the cytoplasm in the control cells, LPS administration led to p65 accumulation in the blue-labeled nuclei (Fig. 6A). However, LPS-enhanced translocation of p65 was prohibited by ISL treatment at 2.5, 5 and 10 μg/mL (Fig. 6B), similar to the effect of DEX (Fig.6A).

The p65 fraction in the nucleus was further detected. As shown in Fig. 6C, LPS stimulation resulted in higher levels of p65 in the nucleus than that of the nontreatment control (*P* < 0.01). However, the LPS-induced nuclear content of p65 was significantly reduced after the administration of 2.5, 5 and 10 μg/mL ISL (*P* < 0.01), supporting the immunofluorescence results.

## Discussion

To the best of our knowledge, this study is the first to demonstrate the anti-inflammatory effect of ISL in MAC-T cells. Bovine mastitis is often involved in bacterial infections during parturition and early lactation in bovine mammary glands [14]. The MAC-T cell line was established by transfection of simian virus-40 large T-antigen into bovine mammary epithelial cells; thus, the cell line has a biological response similar to that of primary cells [15] and is used as a model for studying bovine mammary gland inflammation. To this end, we chose MAC-T cells to clarify their response to ISL in this study.

It has been reported that LPS, the main component of cell membranes in *Escherichia coli*, triggers a comprehensive immune response in MAC-T cells via TLR4/NF-κB and MAPK cascades [16, 17]. Ma et al [13] indicated an inflammatory response of MAC-T cells after stimulation with 1 μg/ml LPS. Additionally, 1 μg/mL LPS markedly increased the mRNA and protein levels of IL-6, IL-1β and TNF-α (Fig. 2) in this study. Accordingly, 1 μg/mL LPS and ISL at 2.5, 5 and 10 μg/mL without cytotoxicity in MAC-T cells (Fig. 1) were chosen.

Numerous studies have shown that ISL can reduce the production of proinflammatory factors, including IL-6, IL-1β and TNF-α, by blocking TLR4 binding to LPS [17–19]. Our data also showed that ISL downregulated the expression of these proinflammatory factors in LPS-stimulated MAC-T cells (Fig. 2), suggesting the anti-inflammatory effect of ISL. Although there is no report involving ISL efficacy in mammary epithelial cells, the anti-inflammatory effects of ISL in oral squamous cell carcinoma and intestinal and retinal epithelial cells [20–23] are consistent with our results in MAC-T cells.

Other studies have shown that ISL can reduce the expression of COX-2 and iNOS in septic mice [11] and HT-29 cells [22]. COX-2 is an inducible enzyme activated by cytokines and growth factors, such as IL-1 and IL-6, while iNOS is a nitric oxide synthetase activated by pathologic stimuli. Induction of COX-2 and iNOS is responsible for the cell levels of cytotoxic mediators prostaglandin (PG) and NO, respectively [24–26]. Hence, both COX-2 and iNOS are considered markers of oxidative stress and inflammation. The finding that ISL downregulated LPS-enhanced expression of COX-2 and iNOS in MAC-T cells (Fig. 3) indicates the antioxidative effect of ISL on bovine mastitis.

It has been well documented that ISL inhibits NF-κB activity in neurological inflammation, hypertensive renal injury, traumatic brain injury and other inflammatory diseases in rats [11, 27, 28]. NF-κB was first discovered in B cell nuclear proteins bound to the kappa enhancer of the immunoglobulin kappa light chain gene, and it is involved in a series of physiological activities, such as cell growth, inflammation and immune response [29, 30]. NF-κB exists as an inactive complex with IκBα in the cytoplasm. Activation of NF-κB leads to IκB phosphorylation by IκB kinase (IKK) and subsequent degradation via ubiquitination, thereby releasing and translocating NF-κB p65 to the nucleus, where the transcription factor promotes transcription of its target genes, including proinflammatory cytokines, iNOS and COX-2 [31]. Our data revealed that ISL increased the abundance of IκB, but lowered the phosphorylation of IκB and p65 in LPS-stimulated cells (Fig. 4 and Supplementary Fig. 2), consequently blocking p65 translocation to the nucleus (Fig. 6 ). Therefore, the reduced expression of LPS-induced TNF-α, IL-6, IL-1β, iNOS and COX-2 can be due to the suppression of NF-κB activity.

MAPKs are a group of serine/threonine protein kinases that can be activated by a variety of stimuli and regulate the phosphorylation of downstream signaling pathways [32, 33]. MAPKs, consisting of p38, ERK and JNK, are involved in a cascade regulating NF-κB-mediated transcription of the proinflammatory cytokines, i.e. IL-6, IL-1β and TNF-α, and inflammatory mediators, such as NO, iNOS, COX-2 and prostaglandin E2 [24, 34, 35]. Previous reports indicated that ISL inhibited MAPK activities in human liver cancer and colitis [36, 37]. Our results demonstrated significant inhibition of ISL on LPS-induced phosphorylation of p38, ERK1/2 and JNK in MAC-T cells (Fig. 5). These results indicate the involvement of ISL in MAPK-mediated anti-inflammatory and antioxidative effects.

In the current study, we revealed that ISL attenuated the inflammatory and oxidative response *in vitro*, but did not investigated the *in vivo* efficacy of ISL. Especially, we have not carried out clinical trials to explore the clinical efficacy of ISL in the treatment of bovine mastitis at present. Nevertheless, findings from previous studies have highlighted the anti-inflammatory and antioxidative activities of ISL via the NF-κB, MAPK or other pathways in rats with carrageenan-induced pleurisy [38] and in mice with dextran sulfate sodium-induced colitis [37]. The above data together with the findings from the present studies will help to determine the potential application of ISL in the prevention and treatment of bovine mastitis.

## Conclusions

ISL downregulats LPS-induced expression of inflammatory cytokines, i.e. TNF-α, IL-1β and IL-6, and oxidative stress indicators, which include iNOS and COX-2, in MAC-T cells. The anti-inflammatory effect of ISL involves the inhibition of the NF-κB and MAPK pathways.

## Methods

### Reagents and chemicals

ISL and dexamethasone (DEX) were provided by Macleans (Shanghai, China). LPS from *Escherichia coli* 055:B5 serotype, purity ≥ 99%, item number: L8880) and MTT [3-(4,5-dimethylthiazol-2-yl)-2,5-diphenyltetrazole bromide] were provided by Solarbio (Beijing, China). The fluorescence quantification kits were purchased from Takara (Beijing, China). Primary antibodies were obtained commercially, including p-p65, p-IκBα, p65, IκBα, p-ERK1/2 and ERK1/2 (Bioss, Beijing, China) as well as JNK, p38, p-JNK and p-p38 (Wanleibio, Shenyang, China). Fetal bovine serum (FBS) and Dulbecco's modified Eagle's medium (DMEM) were supplied by Gibco (Suzhou, China). The ELISA and NF-κB Activation Nuclear Transport Test Kits were available from SinoBestBio (Shanghai, China) and Beyotime (Shanghai, China), respectively. Tris-buffered saline plus Tween 20 (TBST) was obtained from Solarbio (Shanghai, China).

### Cell culture and treatments

MAC-T cells were kept in our laboratory and were seeded at 37°C and 5% CO_2_ in an incubator in DMEM basic, which contained 10% FBS, 100 U/mL streptomycin and 100 μg/mL penicillin. The cells were grown to 90-100% confluency at logarithmic phase, and then the cells were administered with 1 μg/mL LPS combined with 2.5, 5, or 10 μg/mL ISL in five replicates for 24 h. LPS (1 μg/mL) was used according to recent reports [3, 4], and ISL concentrations were determined from the cell viability experiments. Cells in LPS (1 μg/mL) without ISL and in LPS with 20 μg/mL of DEX, a drug for anti-inflammation, were used as negative and positive controls, respectively.

### Cytotoxicity test

Cells were plated in a 96-well plate in 5 replicates and treated with ISL at 0, 2.5, 5, 10, 20 and 40 μg/mL for 24 h. The culture medium was replaced with 10 μL of MTT solution and 90 μL of DMEM, and the cells were incubated for another 4 h. After centrifugal precipitation at 1000 rpm for 5 min, the cells were incubated again in 110 μL of formazan solution for 10 minutes. Measurement of optical density (OD, 490 nm) was performed in xMarkTM (BIO-RAD, California, America).

### Real-time PCR analysis

Cells were seeded in 12-well plates. After extraction of total RNA using the RNAiso Plus, cDNA was synthesized using the Reverse Transcription System (Takara, Beijing, China). Real-time PCRs were performed in triplicate using the TB Green® Premix Ex Taq™ II (Takara, Beijing, China) with the primers in Table 1. The melting curve analysis, PCR cycling parameters and conditions were documented recently [39]. The 2^−ΔΔCt^ method was used to measure the mRNA levels of genes related to the gene of β-actin [13].

**Table 1:** Primer information for real-time PCR

Gene name	Primer Sequence 5′–3′	Size (bp)	Annealing (°C)	Accession No.
TNF-alpha	F: TCTGGTTCAAACACTCAGGTCC	120	59	NM_173966
	R: AGGGCATTGGCATACGAGTC			
IL-1beta	F: AGAGGCAGTTTGGGAGACGA	241	59	NM_174093
	R: GGGACTGGCATGGCAAATGG			
IL-6	F: CACCCCAGGCAGACTACTTC	216	59	NM_173923
	R:AAGCAAATCGCCTGATTGAACC			
iNOS	F: CTGGAGGAAGTGGGCAGAAG	190	59	NM_001076799
	R: CTCGGGAGCGGTACTCATTC			
COX-2	F: TAAAGCCAGGGGAGCTACGA	191	59	NC_006853
	R: TAAGCCTGGACGGGACGATA			
Beta-actin	F: AGCAGATGTGGATCAGCAAG	82	59	NM_173979
	R: TAACAGTCCGCCTAGAAGCA			

### ELISA (enzyme-linked immunosorbnent assay) and endotoxin test

Cells were seeded in a 6-well plate and a 24-well plate for the detection of cytokines and endogenous endotoxin, respectively. Cell protein was extracted and quantified with Total Protein Extraction Kits and then BCA Protein Quantification Kits (Vazyme, Nanjing, China). The contents of IL-1β, TNF-α, IL-6, iNOS and COX-2 were detected by commercial ELISA kits (Youxuan, Shanghai, China) in triplicate, while endogenous endotoxin in cells was determined using Endotoxin (ET) ELISA kits (Jiangsu Jingmei Biological Technology, Yangcheng, China ). The OD values at 450 nm were measured in xMark^TM^ (BIO-RAD, California, America).

### Western blotting analysis

Cells were cultured and treated as described above in a 6-well plate. Western blotting detection of IκBα, p-IκBα, p65, p-p65, p38, p-p38, JNK, p-JNK, ERK and p-ERK was performed as recently described [13]. Each experiment was repeated three times, and the β-actin protein was used as an endogenous control as described in a previous report in MAC-T cells [13]. Band intensity was measured using Image Lab software (version 5.2.1, Bio-Rad, California, America).

### Measurement of p65 nuclear translocation

Immunofluorescence and ELISA assays for p65 nuclear translocation were analyzed as recently described [3, 13]. Briefly, MAC-T cells were cultured in a 6-well plate until 5,000 cells/well. The cells were incubated sequentially with the primary antibody against p-p65 and rabbit anti-goat IgG/Cy3 antibody. The nucleus was stained blue with 4',6-diamino-2-phenylindole (DAPI) and observed under a fluorescence inverted microscope (Leica, Wetzlar GER). For detection of p65 nuclear translocation, nuclear protein was extracted and quantified using a nuclear protein extraction kit and a BCA protein quantification kit (Vazyme, Nanjing, China), respectively. The levels of p65 in the nucleus were measured with commercial ELISA kits as described above.

### Statistical Analysis

Statistical analysis was performed using SPSS 23.0. The data are expressed as the means ± standard error of the mean (SEM). The t-test was conducted between controls and LPS treatments without DEX or ISL. One-way analysis of variance, followed by Tukey’s post hoc test, was used to compare the significance among LPS combined with DEX and ISL treatments. Statistical significance was set at *P* < 0.05 or *P* < 0.01.

## Supplementary Information

**Additional file 1: Supplemental Fig. 1.** LPS-induction of IL-6 mRNA in MAC-T cells. Cells were respectively coincubated with LPS at 1, 5, 10 and 20 μg/mL for 24 h. Real-time PCR was used to measure IL-6 mRNA levels with β-actin as an internal control. Values represent the means ± SEM of four independent experiments. In the above bars, ** indicate significance at *P* < 0.01 between the control and the LPS treatment without DEX and ISL. Among LPS in combination with DEX and ISL treatments, the same letters indicate *P* > 0.05. **Supplemental Fig. 2.** Western blotting analysis of p-p65/p65 and p-IκB/IκB in MAC-T cells. (**A**) Western blotting. (**B and C)** p-p65/p65 and p-IκB/IκB, respectively. Cells were treated with 1 μg/mL lipopolysaccharide (LPS) in combination with dexamethasone (DEX) (20 μg/mL) or ISL (2.5, 5 and 10 μg/mL) for 24 h. The displayed gels were cropped from the original images in the additional files. In the above bars, ** indicate significance at *P* < 0.01 between the control and the LPS treatment without DEX and ISL. Among LPS in combination with DEX and ISL treatments, the same letters indicate *P* > 0.05; different lowercase letters indicate *P* < 0.05 while different uppercase letters indicate *P* < 0.01. **Supplemental Fig. 3.** Western blotting analysis of p-p38/p38, p-ERK/ERK and p-JNK/JNK in MAC-T cells. (**A**) Western blotting. (**B**-**D**) p-ERK/ERK, p-p38/p38 and p-JNK/JNK, respectively. Cells were incubated with 1 μg/mL lipopolysaccharide (LPS) in combination with dexamethasone (DEX) (20 μg/mL) or isoliquiritigenin (ISL) (2.5, 5 and 10 μg/mL) for 24 h. The displayed gels were cropped from the original images in the additional files. In the above bars, ** indicate significance at *P* < 0.01 between the control and the LPS treatment without DEX and ISL. Among LPS in combination with DEX and ISL treatments, the same letters indicate *P* > 0.05; different lowercase letters indicate *P* < 0.05 while different uppercase letters indicate *P* < 0.01.

## Data Availability

The datasets generated and/or analyzed during the current study are available in the figshare repository, https://figshare.com/articles/figure/Anti-inflammation_of_isoliquiritigenin_in_LPS-stimulated_MAC-T_cells/19067642.

## Abbreviations

ISL: Isoliquiritigenin
NF-κB: Nuclear factor kappa B
TLR4: Toll-like receptor 4
MAPK: Mitogen-activated protein kinase
LPS: Lipopolysaccharide
MAC-T: Bovine mammary epithelial cells
IKK: IκB kinase
IL-1β: Interleukin-1β
TNF-α: Tumor necrosis factor-α
ERK: Extracellular signal-regulated kinase
JNK: C-jun NH2 terminal kinase
IL-6: Interleukin-6
COX-2: Cyclooxygenase-2
iNOS: Inducible nitric oxide synthase
