# Supplementary material for: Anti-inflammation of isoliquiritigenin via the inhibition of NF-κB and MAPK in LPS-stimulated MAC-T cells
Source: BMC Vet Res. 2022 Aug 19;18:320. doi: 10.1186/s12917-022-03414-1 (PMC9392288; doi:10.1186/s12917-022-03414-1)
Supplement: Supplementary file 1 — Additional file 1: Supplemental Fig. 1. LPS-induction of IL-6 mRNA in MAC-T cells. Cells were respectively coincubated with LPS at 1, 5, 10 and 20 μg/mL for 24 h. Real-time PCR was used to measure IL-6 mRNA levels with β-actin as an internal control. Values represent the means ± SEM of four independent experiments. In the above bars, ** indicate significance at P < 0.01 between the control and the LPS treatment without DEX and ISL. Among LPS in combination with DEX and ISL treatments, the same letters indicate P > 0.05. Supplemental Fig. 2. Western blotting analysis of p-p65/p65 and p-IκB/IκB in MAC-T cells. (A) Western blotting. (B and C) p-p65/p65 and p-IκB/IκB, respectively. Cells were treated with 1 μg/mL lipopolysaccharide (LPS) in combination with dexamethasone (DEX) (20 μg/mL) or ISL (2.5, 5 and 10 μg/mL) for 24 h. The displayed gels were cropped from the original images in the additional files. In the above bars, ** indicate significance at P < 0.01 between the control and the LPS treatment without DEX and ISL. Among LPS in combination with DEX and ISL treatments, the same letters indicate P > 0.05; different lowercase letters indicate P < 0.05 while different uppercase letters indicate P < 0.01. Supplemental Fig. 3. Western blotting analysis of p-p38/p38, p-ERK/ERK and p-JNK/JNK in MAC-T cells. (A) Western blotting. (B-D) p-ERK/ERK, p-p38/p38 and p-JNK/JNK, respectively. Cells were incubated with 1 μg/mL lipopolysaccharide (LPS) in combination with dexamethasone (DEX) (20 μg/mL) or isoliquiritigenin (ISL) (2.5, 5 and 10 μg/mL) for 24 h. The displayed gels were cropped from the original images in the additional files. In the above bars, ** indicate significance at P < 0.01 between the control and the LPS treatment without DEX and ISL. Among LPS in combination with DEX and ISL treatments, the same letters indicate P > 0.05; different lowercase letters indicate P < 0.05 while different uppercase letters indicate P < 0.01. [file 12917_2022_3414_MOESM1_ESM.docx]

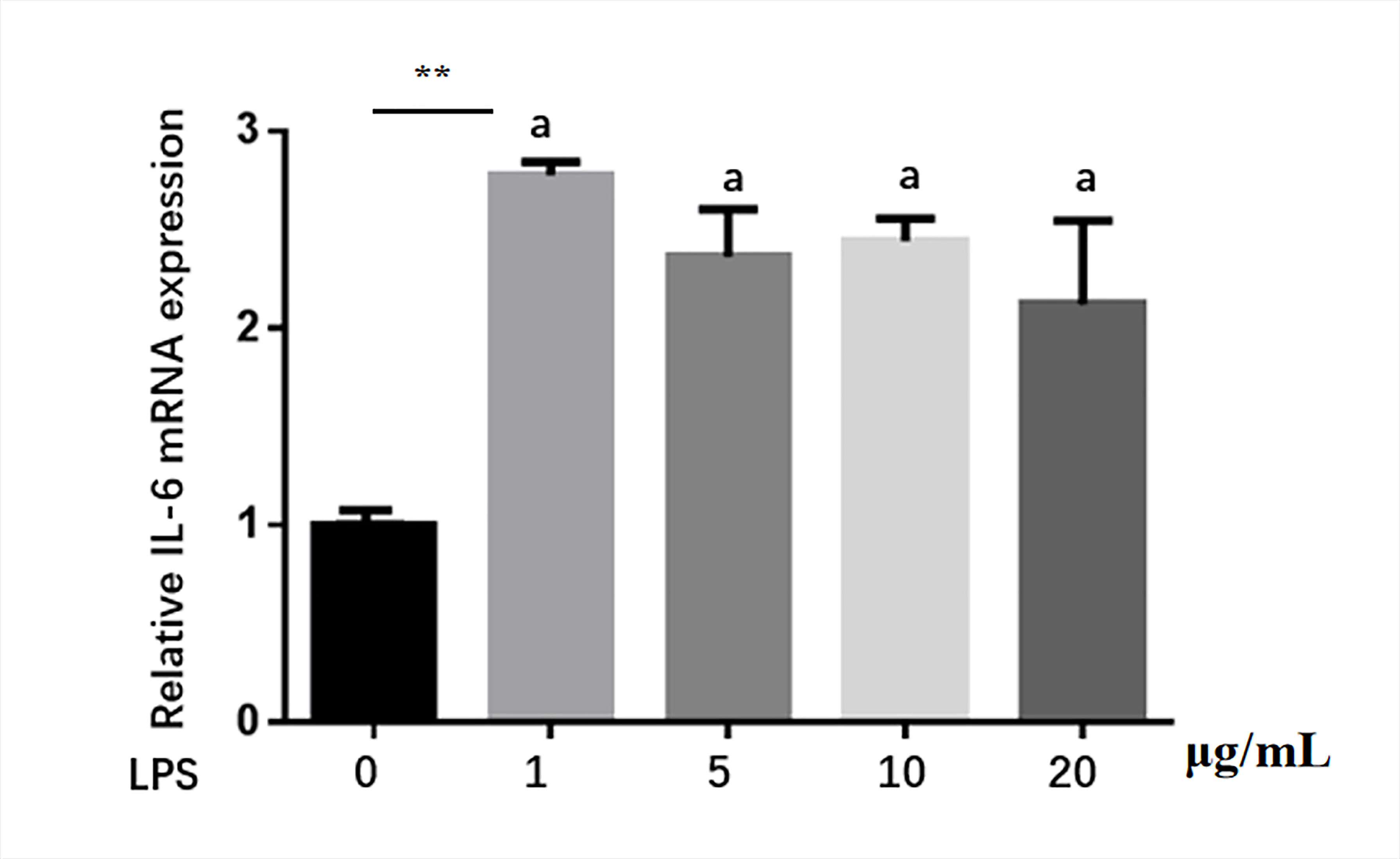


**Supplemental Fig.1** LPS-induction of IL-6 mRNA in MAC-T cells. Cells were respectively coincubated with LPS at 1, 5, 10 and 20 µg/mL for 24 h. Real-time PCR was used to measure IL-6 mRNA levels with β-actin as an internal control. Values represent the means ± SEM of four independent experiments. In the above bars, ** indicate significance at *P* < 0.01 between the control and the LPS treatment without DEX and ISL. Among LPS in combination with DEX and ISL treatments, the same letters indicate *P* > 0.05.


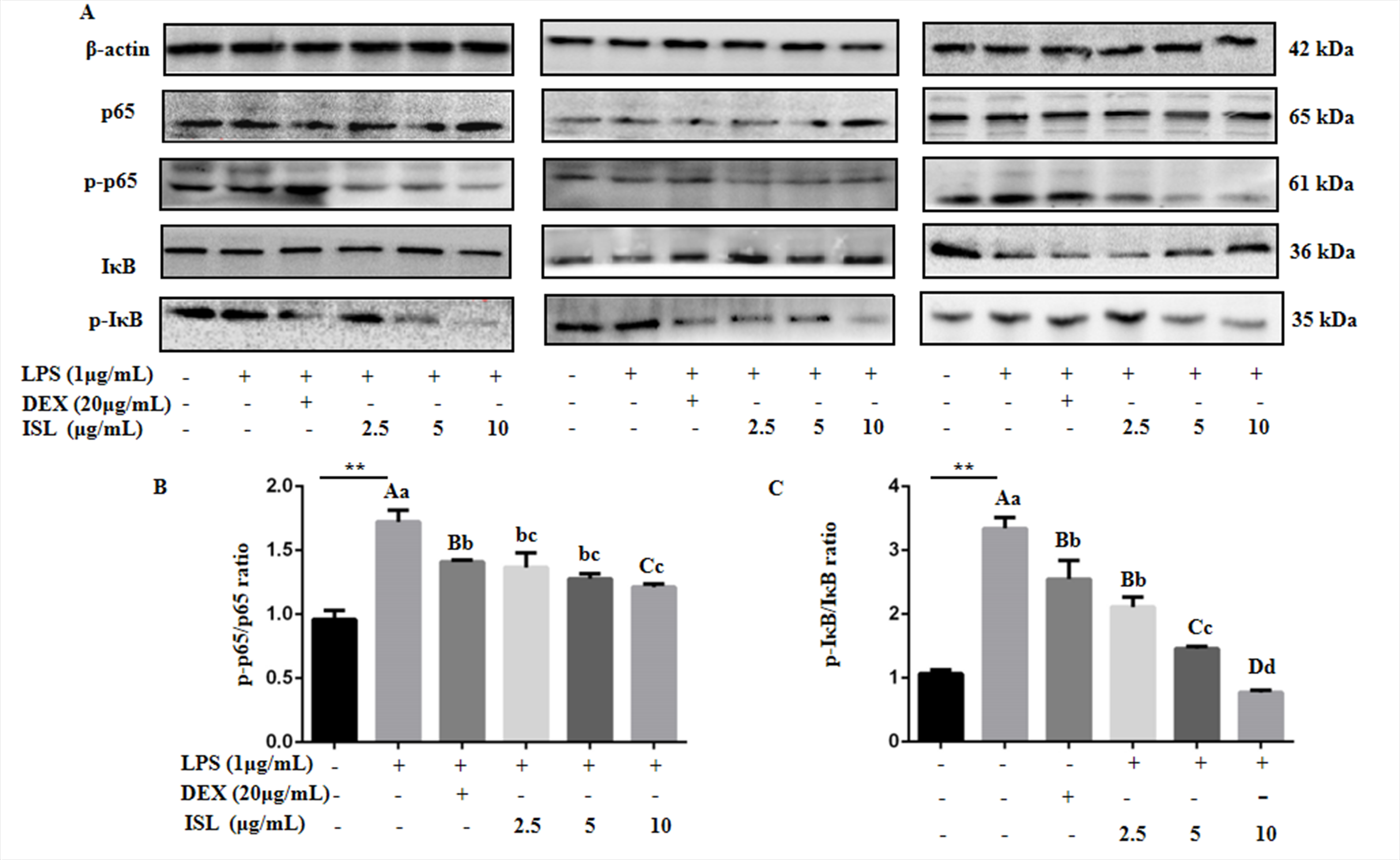


**Supplemental Fig. 2** Western blotting analysis of p-p65/p65 and p-IκB/IκB in MAC-T cells. (**A**) Western blotting. (**B and C)** p-p65/p65 and p-IκB/IκB, respectively. Cells were treated with 1 µg/mL lipopolysaccharide (LPS) in combination with dexamethasone (DEX) (20 µg/mL) or ISL (2.5, 5 and 10 µg/mL) for 24 h. The displayed gels were cropped from the original images in the additional files. In the above bars, ** indicate significance at *P* < 0.01 between the control and the LPS treatment without DEX and ISL. Among LPS in combination with DEX and ISL treatments, the same letters indicate *P* > 0.05; different lowercase letters indicate *P* < 0.05 while different uppercase letters indicate *P* < 0.01.


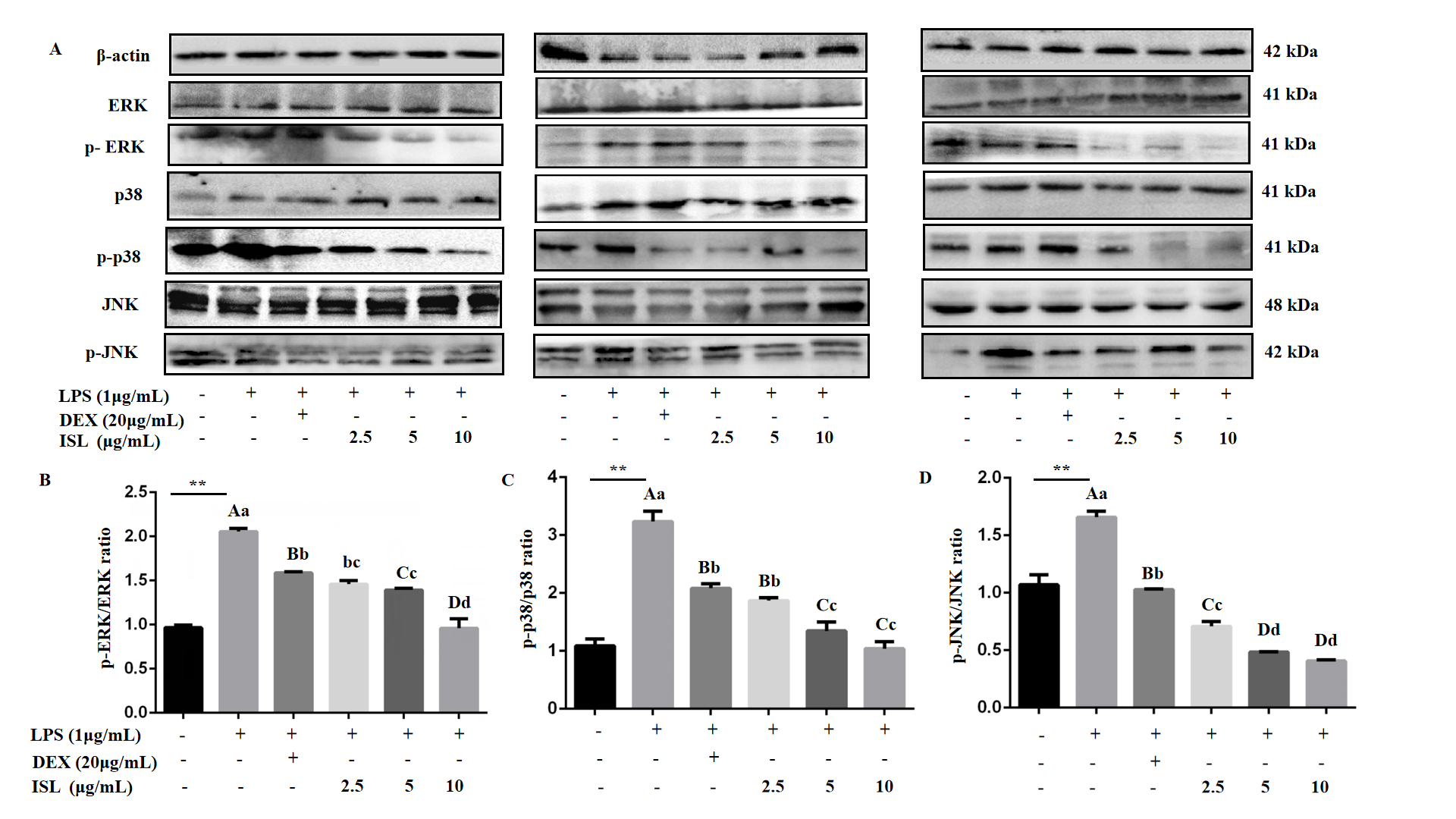


**Supplemental Fig. 3** Western blotting analysis of p-p38/p38, p-ERK/ERK and p-JNK/JNK in MAC-T cells. (**A**) Western blotting. (**B**-**D**) p-ERK/ERK, p-p38/p38 and p-JNK/JNK, respectively. Cells were incubated with 1 μg/mL lipopolysaccharide (LPS) in combination with dexamethasone (DEX) (20 μg/mL) or isoliquiritigenin (ISL) (2.5, 5 and 10 μg/mL) for 24 h. The displayed gels were cropped from the original images in the additional files. In the above bars, ** indicate significance at *P* < 0.01 between the control and the LPS treatment without DEX and ISL. Among LPS in combination with DEX and ISL treatments, the same letters indicate *P* > 0.05; different lowercase letters indicate *P* < 0.05 while different uppercase letters indicate *P* < 0.01.
